# Importance and implications of exosomes in nephrology and urology

**DOI:** 10.1007/s00424-022-02771-y

**Published:** 2022-11-18

**Authors:** Lina Mahl, Johanna Ollig, Verena Schweihofer, Lara Wiegand, Phillipp Torkler, Silke Haerteis, Thiha Aung

**Affiliations:** 1 Institute for Molecular and Cellular Anatomy, University of Regensburg, 93053 Regensburg, Germany; 2 Faculty of Computer Science, Deggendorf Institute of Technology, 94469 Deggendorf, Germany; 3 Faculty of Applied Healthcare Science, Deggendorf Institute of Technology, 94469 Deggendorf, Germany

**Keywords:** Exosomes, Extracellular vesicles, Nephrology, Urology, Uro-oncology

## Abstract

Exosomes are extracellular vesicles that are formed by two invaginations of the plasma membrane and can be released by all eukaryotic cells. Because of their bioactive contents, including nucleic acids and proteins, exosomes can activate a variety of functions in their recipient cells. Due to the plethora of physiological and pathophysiological functions, exosomes have received a lot of attention from researchers over the past few years. However, there is still no consensus regarding isolation and characterization protocols of exosomes and their subtypes. This heterogeneity poses a lot of methodical challenges but also offers new clinical opportunities simultaneously. So far, exosome-based research is still mostly limited to preclinical experiments and early-stage clinical trials since the translation of experimental findings remains difficult. Exosomes could potentially play an important role as future diagnostic and prognostic agents and might also be part of the development of new treatment strategies. Therefore, they have previously been investigated in a variety of nephrological and urological conditions such as acute kidney injury or prostate cancer.

## Introduction

**Fig. 1:**
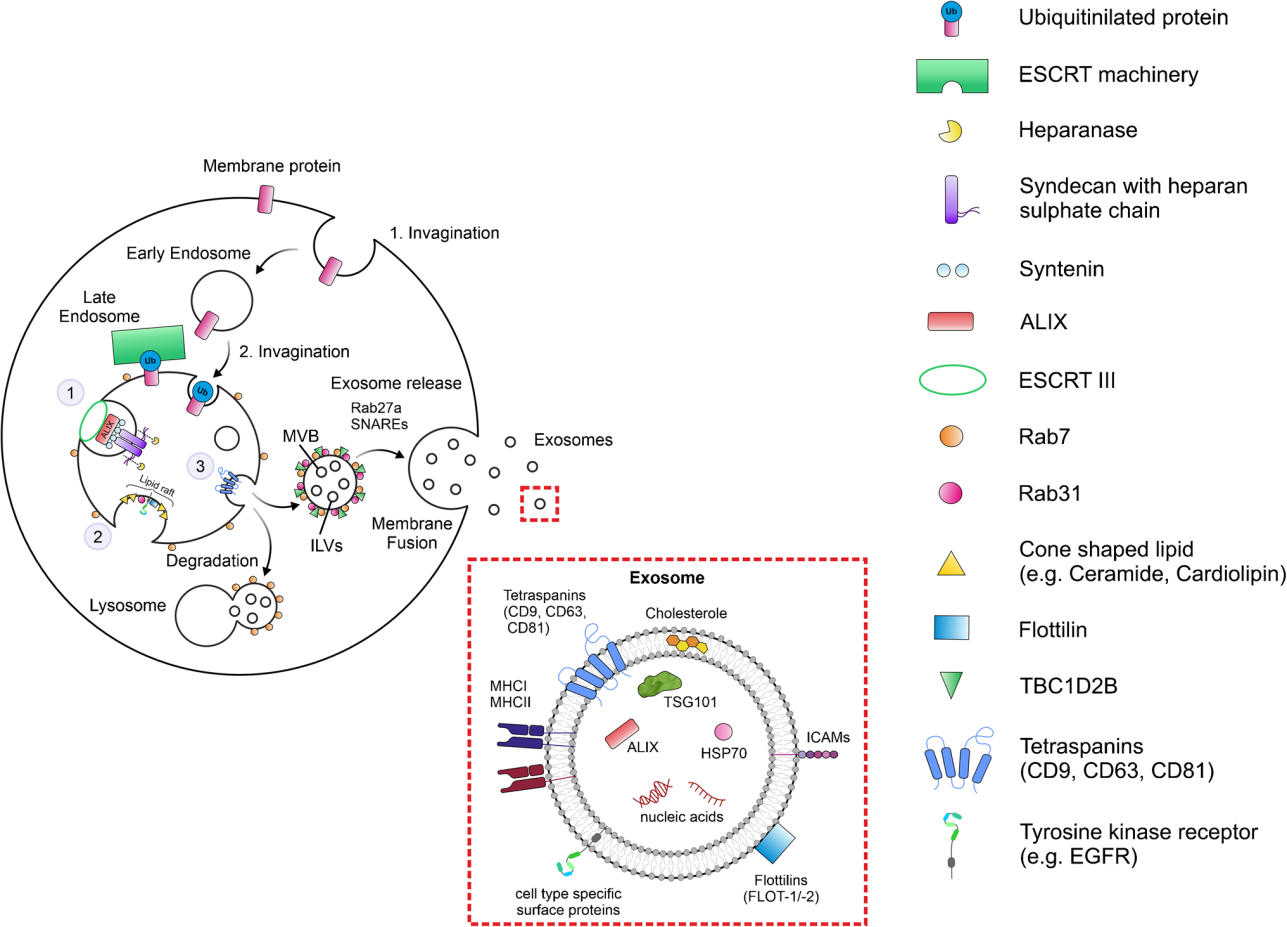
Biogenesis of exosomes: (1) ESCRT-dependent pathway: ESCRT 0-III work closely together to facilitate the second budding step and sort ubiquitin-tagged proteins into the ILVs. They are assisted by the syndecan-syntenin-ALIX adapter complex, which stabilizes ESCRT III at the neck of the vesicle. Finally, ESCRT III mediates the sequestration of the vesicles into the lumina of the endosomes. (2) ESCRT independent pathway: Budding is mainly mediated by a modified lipid composition of the vesicle membrane, with ceramides, cardiolipids, or cholesterol. Flotillins and Rab31 help to internalize tyrosine kinase receptors besides acting as scaffold proteins and preventing lysosomal degradation, respectively. (3) A third major contribution is provided by tetraspanins: they mediate the sorting of various proteins into the ILVs by forming of microdomains. The image was created with CorelDRAW Graphics Suite (Corel, Ottawa, ON, Canada)

Extracellular vesicles (EVs) are membrane-enclosed, vesicular structures that can be subdivided into three major categories based on their size and biogenesis: apoptotic bodies, microvesicles, and exosomes [71]. Exosomes are the smallest EVs with a diameter of approximately 40 to 160 nm. They are formed by inward-budding of endosomes and are released from multivesicular bodies (MVB) by the vast majority of eukaryotic cells [45, 71, 110]. Like most EVs, exosomes play a major role for intercellular communication and contain bioactive contents composed of proteins, lipids, and nucleic acids [65, 66, 72, 84, 96]. Some proteins (CD9, CD63, CD81) are widely expressed among all exosomes, but the identification of specific exosomal marker proteins remains difficult because a majority of essential proteins for formation and secretion of exosomes (see Fig. 1) can be found among all EVs [47, 66, 75]. Additionally, the composition of exosomes is dependent on the cell of origin and its current state. Consequently, the secreting cell is able to elicit a variety of cell-specific functions in its target cells, including physiological and pathophysiological effects for example in different cancers or renal diseases [109, 110].

The heterogeneous content of exosomes, their high stability, the protection of the cargo through a liquid bilayer, and their function in intercellular communication has led to a strong interest in pursuing their utilization for diagnosis, prognosis, carriers of vaccines, and therapy of different diseases with a large focus on cancer [56, 81, 109].

The procedures required for the isolation and proliferation of exosomes, along with their bioactive and cell-specific components are important steps for the characterization of exosomes and their potential diagnostic and prognostic value [109]. Several different methods and protocols for the isolation, separation, proliferation, and characterization of exosomes have led to insufficiently supported conclusions concerning the functions and applications of EVs. To improve reliability, reproducibility, and acceptance of EV research, the International Society for Extracellular Vesicles (ISEV) published updated Minimal Information for Studies of Extracellular Vesicles (MISEV) guidelines in 2018. These guidelines aim at implementing a methodical standard for the acquisition of EV- and exosome-related data to enable a sufficient interpretation of the acquired results [94].

In this review, we provide an overview on the current state of research on exosomes and their medical applications in nephrology and urology. Due to the afore mentioned difficulty in assigning specific functions and features to EV subtypes, ISEV has recommended to stop using terms such as exosomes which are associated with sometimes contradictory findings [78]. Despite these difficulties, the terms exosomes and EVs were used cautiously and on the basis of the descriptions of the cited works.

## Background: structure and biogenesis

Exosomes are generated by two invaginations: First, endosomes are sequestered from the plasma membrane. Once matured to late endosomes, they release so-called intraluminal vesicles (ILVs) into the lumina, which become multivesicular endosomes or multivesicular bodies (MVBs). Subsequently, there are two different pathways for the further development: either they merge with lysosomes or autophagosomes, causing the degradation of their content, or they merge with the plasma membrane, releasing their entrapped ILVs into the extracellular space as exosomes (Fig. 1). This whole process is accompanied by the constant restructuring of both the membrane components (lipids, surface proteins) of the respective vesicles and their contents including proteins, nucleic acids, and metabolites. Overall, the lipid composition of the exosomal membrane largely consists of membrane lipids and has been described as relatively similar amongst exosomes of different cellular origin but varies from the composition of the parental cell.

Although the endosomal origin of exosomes has been generally acknowledged for several decades, the molecular mechanisms of exosomal biogenesis are still unclear. Even though many details have not been clarified yet, researchers have still agreed on two possible pathways of biogenesis: an ESCRT (endosomal sorting complex required for transport)-dependent pathway and an ESCRT-independent pathway, mediated by tetraspanins and several lipids (Fig. 1) [7, 75, 103]. The existence of the latter and its role in the case of dysfunction of the ESCRT machinery has been proven by siRNA-induced depletion of various ESCRT key elements by Stuffers et al. [92].

The coexistence of these two pathways at the cellular level and how much each mechanism contributes proportionally to ILV formation is likely cell type specific [37] and is supposed to generate pathway-depending MVB subtypes [92]. So far, it is believed that there is a close interplay of ESCRT-dependent and independent mechanisms, enabling cells to secrete exosomes under variable cellular conditions. The secretion and specific uptake of exosomes which are essential for the intercellular communication via exosomes are believed to be regulated by intersecting pathways as well.

## Methods for the isolation of EVs, included exosomes

In accordance with the MISEV guidelines of the ISEV from 2018, the purification of exosomes from collected bio-fluids and the separation from non-EV components and other EV-types can be performed by various methods. These include ultracentrifugation (UC), precipitation methods (e.g. polyethylene glycol (PEG)-based), density gradient centrifugation (DGC), size exclusion chromatography (SEC), and immunomagnetism, among others [94]. According to two worldwide surveys from 2015 and 2019, separation via ultracentrifugation (UC) is the most frequently used method but alternative separation techniques increased from 2015 to 2019 (Table 1) [32, 78]. Recent findings revealed a strong influence of separation methods on subsequent RNA analyses and high method-to-method variations. Ideally, exosome separation methods are selected based on the target bio-fluid and molecular analyte [23, 70, 90]. In addition to the MISEV guidelines, the Urine Task Force of ISEV presents the current state of the art as well as current challenges in urinary EV analyses for clinical applications in great detail [25].

**Table 1:** Summary of isolation and purification methods. Methods are summarized in accordance with the MISEV2018 guidelines for exosome-based research based on their use in 2019, their costs, the time needed for analysis, amd the purity, specificity, complexity, and outcome (recovery and functionality of exosomes) of the procedure (adapted from [83]). UC = ultracentrifugation; SEC = size exclusion chromatography; DGC = density gradient centrifugation; PEG = polyethylene glycol-based precipitation methods; IM = immunomagnetism; MF = microfluidics; 1: + (low); +  + (intermediate); +  +  + (high). 2: + (no); +  + (yes); NS = tendency from 2015 to 2019 not significant; * depending on kit; ** combination with DGC/SEC: +  +  + ; *** shear forces may affect functionality

Method	Usage (2019), Tendency	Cost^1^	Need of time^1^	Recovery^1^	Purity^1^	Specifity^1^	Complexity^1^	Functionality of Evs^2^
UC	75%, ↘^NS^	+	+ + +	+	+ + **	+ +	+ +	+ + ***
SEC	~ 40%, ↗	+	+	+ + +	+ +	+ +	+	+ +
DGC	~ 35%, ↗	+	+ +	+ +	+ +	+ +	+	+ +
PEG	~ 25, ↗	+	+ / + + *	+ + +	+	+	+	+ +
IM	~ 20%, ↗	+ +	+ +	+ +	+ + +	+ + +	+ +	+ +
MF	4%, ↗	+ + +	+ + +	+	+ + +	+ + +	+ + +	+

Besides total isolation of EVs and exosomes from biofluids, specific isolation methods for tumor EVs are investigated. Recent models of EV kinetics suggest that bulk EV measurements are limited in their sensitivity to detect small tumors due to low signal-to-noise ratios from small tumors [27]. To increase the signal from tumor derived exosomes, several approaches to enrich, deplete, and filter EVs are under active development. This may result in improved early diagnostic approaches in comparison to diagnostics based on bulk measurements [28, 71, 73].

### Preparation of fluids

According to the current MISEV guidelines, extracted fluids containing the targeted EVs have to be pre-cleaned (centrifugation and/or filtration) to remove remaining cells and cellular debris [94]. After pre-cleaning of the supernatant, a protease inhibitor can be added to prevent protein degradation [20]. For EDTA-blood samples (or other body fluids like cerebrospinal fluid), a second centrifugation step should be implemented to remove platelets and remaining white blood cells [16]. Due to the high concentration of uromodulin (Tamm-Horsfall protein) in urine, some uromodulin-bound EVs are included in the low-speed pellet after centrifugation and not in the high-speed pellet. They can be released by the reduction of disulfide-bonds of uromodulin through treatment with reducing agents (e.g., Dithiothreitol) [29].

### Isolation and purification methods

#### Centrifugation-based methods

UC can be used for the isolation of EVs from all fluids (cell culture supernatants and all body fluids), and control fractions can be used as negative controls [16]. The limitations of UC include the fact that it is a time-intensive procedure which is restricted to the processing of minor volumes and the impact of centrifugal force on the vesicles. Furthermore, portions of the sample can be lost or contaminated with particles of similar density and size with limited reproducibility. A combination with DGC, SEC, or filtration is recommended to avoid contamination [16, 26]. Brennan et al. reached the highest ratio of plasma-derived EVs with 61–150 nm/particles with 0–60 nm with a combination of DGC and UC. However, this combination also had the highest level of unwanted APO-B-containing lipoproteins [9]. Because of the above-mentioned disadvantages and the high costs of UC, other methods are needed, for example, DGC itself where EVs are isolated with a sucrose density gradient. This methodology is especially well suited for the extraction of EVs with low content [57], but contamination of the targeted fractions with particles of similar density is still possible.

#### Precipitation and size exclusion-based methods

Another method is the precipitation of less soluble components by introducing water-excluding polymers such as PEG or lectins to the samples and the isolation of precipitated EVs which are unable to solubilize with subsequent centrifugation or filtration. This enables the isolation of EVs from large sample sizes and represents the basis of a variety of commercial isolation kits (e.g., ExoQuick ULTRA by System Biosciences) [89]. Furthermore, this method can process around 2.5-fold higher concentrations than UC and uses no centrifugal forces while being easily reproducible [16, 26] and has been used increasingly in recent years (Table 1). Setbacks of this method include the number of impurities (e.g., IgG or Albumin which are likely bound to exosomes) [16] and the nonspecific isolation of vesicles (particles with sizes > 150 nm and < 60 nm) [9]. Alternatively size exclusion methods which rely on the separation of EVs based on their size can be used. SEC, which divides the particles by size using porous polymer microspheres, has gained a lot of attention [89]. It enables the division of the different vesicle classes into distinct fractions [16] and can be performed afterwards for the exclusion of remaining contaminants (e.g., in high-performance liquid chromatography (HPLC)).

#### Novel isolation methods

Immunoaffinity/-magnetic methods (e.g., MACS-Separation) are also promising because they are easy to use and EVs are not subjected to centrifugal or chemical forces (Miltenyi Biotec B.V. & Co. KG, Bergisch Gladbach, Germany). They require the definition of EV-specific membrane-bound biomarkers that can be targeted by antibodies and in the case of immunomagnetic isolation immune-magnetic beads that are coated with antibodies and can be separated from the structure of interest by a magnetic field [89]. Currently, there are only three antibodies available for this purpose which highlights the need for identification of more specific exosomal targets and tissue-specific EV targets. Other novel methods include microfluidics (combination of immunoaffinity via antibody-linked binding and membrane filtration) which are often costly and can lead to a loss of function in EVs [83] (Table 1) or EXODUS (exosome detection via the ultrafast-isolation system) which was established as a new method of ultracentrifugation. It is based on the coupling of two oscillators, which set the nanoporous membranes of the filter system into high-frequency vibration by generating transverse waves. This vibration prevents clogging of the filter, which gives EXODUS a considerable advantage over conventional filtration methods. Furthermore, EXODUS is significantly less time-consuming than other methods such as ultracentrifugation or PEG precipitation and provides better results in terms of yield and purity, as Chen et al. were able to demonstrate using urine samples [15]. A final isolation method that should be mentioned here is asymmetric flow field-flow fractionation (AF4). This method makes use of the different hydrodynamic properties of macromolecules for separation. A laminar flow is generated, which separates the analytes diffusing freely through a semi-permeable membrane according to their size. The major difference to conventional chromatographic methods is the absence of a stationary phase, which means that the analytes are not exposed to any of the known risks caused by binding to the stationary phase or shear forces. Although this method has been known for several decades, AF4 was only established for exosome isolation in 2019 by Zhang et al. AF4 is characterized by its good reproducibility, as well as its flexibility as the precise isolation is not dependent on specific surface molecules. It is also favored because of its shorter processing time and the lack of contamination by smaller molecules [107].

In conclusion, there is a mix of different isolation methods that display advantages and disadvantages with no standardized isolation procedure. Choosing the right method should be based on the type and volume of fluid that EVs are isolated from as well as on the design of the study. To utilize EVs in a clinical setting (e.g., as biomarkers), several challenges such as the isolation of different subtypes have yet to be overcome [97]. At the moment, methods for EV isolation are optimized accordingly, and new possibilities such as flow cytometry are being explored as alternative procedures [89].

### Characterization and analysis

To ensure a successful isolation, the isolated vesicles should be visualized, quantified, and analyzed. For the visualization, identification of EVs and assessment of their integrity, either single EV particles or their content, can be analyzed.

#### Microscopic methods

Microscopic analyses such as transmission electron microscopy (TEM), cryo-EM, and other methods (scanning-probe microscopy (SPM) including atomic force microscopy (AFM)) [94] not only enable the visualization of EV morphology but can depict mechanisms of EV uptake and secretion as well. EV imaging also includes fluorescent imaging, which is useful for visualization within cells, cell groups and tissues (e.g. NanoImager, fluorescence microscopy) [25] (Keyence GmbH, Neu-Isenburg, Germany). Other clinical approaches include magnetic resonance imaging (MRI) in combination with nanoparticle-labelled exosomes [10], which can be used for the visualization of the distribution of exosomes. Some of these methods require the preparation or fixation of the samples, which has been argued to possibly change the morphology of EVs, which is why new microscopic technologies such as cryo-EM or atomic force microscopy (AFM) are supposed to be introduced for EV characterization [89]. Until then, nanoparticle tracking analysis (NTA) is amongst one of the most popular methods, which can be used for determining the size distribution (as it is very accurate when it comes to the measurement of size (Malvern Panalytical Ltd, Malvern, UK)) as well as the concentration of isolated EVs based on laser measurements of the scattered light of EVs in solution [16, 89]. However, NTA cannot distinguish between exosomes and other similar vesicles, which is why the concentration can be overestimated.

#### Identification of proteins, metabolites, and lipids

In comparison, flow cytometry, which enables the examination of the heterogeneity of the surface protein expression, is characterized by its ability to distinguish different (sub-)types of analytes. It allows the identification of single particles based on their fluorescent signals which are generated by a laser beam as they are passing through a small nozzle [89]. Here, no other vesicles are detected due to targeted immunostaining [16] which can be regarded as the biggest disadvantage, similar to immunomagnetics. Due to the low number of specific antibodies available as well as the low sensitivity caused by the small size of EVs [89], presumably not all subpopulations of EVs can be detected.

The most common, cost-, and time-saving method to identify isolated EVs and to analyze the different fractions of DGC or SEC is the western blot (WB) [33]. It can be used as an Immunoblot to identify unwanted contaminants, for example by targeting apolipoproteins (e.g., APO-E, APO-B) with antibodies to detect lipoprotein vesicles [9]. Another possible application is the so-called SDS-PAGE (sodium dodecyl sulfate polyacrylamide gel electrophoresis) to analyze the entire profile of the sample and determine the distribution of the components like the uromodulin-linked capture of exosomes in urine samples [29].

Mass spectrometry has also been used for the analysis of EV composition regarding proteomics (cytosolic and surface proteins) [77], metabolomics, and lipidomics. As an alternative, chromatography (e.g., HPLC) or Raman spectroscopy, which generates specific protein or metabolite profiles based on laser refraction [80], can be used for lipidomics and metabolomics [16]. To improve the outcome and to find more tissue-specific exosomal surface markers, it is possible to use a combination of existing methods as performed by Wu et al. who developed a proximity-dependent barcoding assay (PBA) which combines antibody-specific binding, rolling circle amplification, PCR, and sequencing of the products [20].

#### RNA analyses

Other approaches include RNA analyses such as microarrays, real-time PCR (RT-PCR), or sequencing of exosomal long-noncoding RNAs (exo-lncRNAs), micro-RNAs (exo-miRs), circular RNAs (exo-circRNAs), and mRNAs (exo-mRNAs) [12, 85, 99]. According to the ISEV guidelines, proteinase or RNAse treatments should be done before the isolation of exosomal RNA (exo-RNA) [89, 94] to remove nucleic acids that are bound on the outside of EVs as well as protein complexes [30]. So far, it is still unknown whether RNAs such as ribosomal RNA (rRNA) or lncRNA fragments which are associated with EVs are artifacts of the isolation process or of biological relevance [30]. There are a few databases such as the exoRBase 2.0 database [50] or the exRNA Atlas resource [69] that summarize exosomal RNA profiles as they can differ depending on the physiological state of the cell [89]. So far, the ISEV guidelines give no specific recommendations for the use of nucleic acids as markers for EVs and see a need for more studies regarding this aspect [94].

Even though there is a significant effort to enable the characterization of exosomes, there is still no effective protocol. This is not only due to the heterogeneity of EVs but also to the varieties within the exosome subclasses [45]. Outcomes in this aspect also depend on the technique used for the preceding isolation of EVs. Nevertheless, methods for EV characterization are essential for understanding EV-related processes and identifying biomarkers in physiological as well as pathophysiological conditions.

## Pathophysiology in different urological and nephrological organ systems

### General oncological and immunological mechanisms

So far, there is a consensus on the assumption that exosomes are involved in the modulation of the immune system, the activation of the complement cascade, and also in pathophysiological processes such as multi-drug resistance (MDR) in cancer [42, 112]. Hereby, the functional heterogeneity of exosomes depends on the cell or tissue of origin, which in part determines the different sets of their contents [45, 112]. For example, natural killer cells (NK cells) release EVs that contain cell-destructive proteins (FasL, perforins) and tumor-suppressing non-coding RNA (miR-186). On the contrary, EVs secreted by antigen presenting cells (APCs), such as dendritic cells (DCs) and proinflammatory M1-macrophages, contain immunostimulatory proteins like MHC-I and MHC-II [42].

Tumor-derived exosomes, also known as oncosomes, have been extensively studied and promote tumor progression through activation of tumor-promoting pathways, the downregulation of tumor suppressor genes, next to other possibilities [41, 104]. For example, Chen et al. showed that melanoma-derived EVs exhibit programmed death ligand 1 (PD-L1) that interacts with PD-1 of T-cells [34] and inhibits anti-tumor immunity [14].

By transporting bioactive non-coding RNAs (ncRNAs) that induce an enhanced expression of ATP-binding box (ABC) transporters or by direct transport of ABC transporters, tumor-derived exosomes can also induce MDR [104, 112]. ABC transporters lead to an increased efflux of chemotherapeutics in chemosensitive cells and are potentially involved in the biogenesis and spontaneous as well as antigen-triggered release of MDR-inducing EVs [6, 46, 104].

Besides tumor cells, other cells of the tumor microenvironment (TME) are also supposed to be involved in tumor progression by secretion of exosomes. Zhang et al. described the secretion of EVs by M2-subtype macrophages which are also known as tumor-associated macrophages (TAMs) and promote metastasis of different tumor entities by intercellular transmission of tumorigenic agents [111].

Multiple studies have investigated exosomes not only as therapeutic target structures but also as cancer vaccines in immunotherapy due to their biocompatibility, as well as the high stability. Most studies investigating this topic have deployed exosomes derived from dendritic cells (DEXs) either as carriers for anti-tumor agents such as peptides or as effectors after stimulation of DCs with specific cancer biomarkers. There are still challenges in the manufacturing of exosomes, such as the lack of a cell-free platform for EV production and of sufficient quality controls [81]. Multiple reviews highlighted the use of EVs as drug delivery systems and summarized current findings and challenges in detail [38].

### Benign nephrological and urological conditions

Because exosomes mediate a variety of functions, they have been investigated for multiple diseases and conditions using different isolation and characterization procedures. These include nephrological and urological conditions.

Early detection of kidney diseases is still a pressing issue today, as most common biomolecular markers for kidney disease have been described as insensitive and ineffective for an early diagnosis [44]. As methods for early diagnosis and therapy are challenging, end-stage renal disease (ESRD) is still associated with high morbidity and mortality. In recent years, exosomes have emerged as a new approach to solving this problem. Exo-mRNA offers a valuable alternative to RNA extracted from cells out of urine because of its high stability [24]. The close link between EVs and the development and progression of renal diseases could also offer a new therapeutic approach. Due to the size of EVs, it is assumed that urinary EVs originate from tissue that is part of the urinary tract such as the nephron or the urothelium of the bladder and ureter.

#### Diagnostic and prognostic value of exosomes in nephro- and urological conditions

As described above, exosomes play an important role in mediating the immune response and can also contribute to dysregulation of inflammatory reactions. Inflammation which can be triggered by many mechanisms (e.g., ischemia/reperfusion (I/R), proteinuria, toxins) is a central component of acute kidney injury (AKI). Lv et al. demonstrated that increased levels of miR-19b-3p, a negative regulator of a NFκB pathway suppressor, in exosomes derived from injured tubular epithelial cells (TECs) triggered the polarization of macrophages into the M1 proinflammatory phenotype. They investigated kidney and tubular exosomes derived from an AKI mouse model, which they isolated and identified using differential centrifugation as well as UC and a combination of TEM and NTA respectively, as well as exosomes of immortalized mouse TECs in in vitro experiments [62].

So far, the diagnosis of AKI comes along with an increase in serum creatinine or a decrease in urine output, but several exosomal biomarkers have already been identified in urine samples. These include exosomal fetuin-A [113], the transcription factor AFT3 and its mRNA, and a decrease in AQP-1 [5, 85, 86]. Sonoda et al. conducted a longitudinal study, where they identified a strong increase of the expression of miR-200 family members (e.g., miR-141-3p, miR-200a-3p) 3 days after AKI injury. They extracted urinary exosomes and RNAs of IR mouse models using the Urine exosome purification and RNA isolation (Norgen Biotek) as well as a total exosome isolation reagent from urine (Thermo Fisher) and the miRNeasy (Qiagen). Sonoda et al. performed RT (reverse transcription)-PCR for the identification of extracted exosomes. Most of the identified miRNAs regulated an effector of the TGF-β1 pathway which has a direct impact on renal fibrosis [85].

Fibrosis is another pathomechanism that commonly occurs in kidney diseases. Wang et al. studied the change of composition of secreted exosomes under physiological, inflammatory, and fibrotic conditions in primary human proximal TECs. Interestingly, miRNAs (e.g., miR-200a, miR-204), that showed the most alterations under pathophysiological conditions like renal fibrosis, were already known to be elements of renal disease such as progressive CKD. Similarly, many of the abnormally expressed exosomal proteins are involved in pathways of renal fibrosis (e.g., laminin, plectin, fibronectin) or counter-mechanisms such as complement factor C3 or HSPG2 (counteract hypoxia by increasing angiogenesis and vascular permeability) [101]. Other studies defined urinary exo-miR-21, miR-29c, and miR-200b as biomarkers for renal fibrosis [61, 106].

Besides biomolecular markers that indicate renal fibrosis or inflammation, exosomal contents have also been studied regarding their correlation with the progression of CKD. Ding et al. used cell culture and mouse models to show that treatment with isolated EVs of PKD1-cells and urinary EVs from autosomal dominant polycystic kidney disease (ADPKD) patients which were extracted using UC and filtration methods as well as characterized via WB analyses activated the proliferation and formation of cyst-like structures. The promotion of cyst growth was attributed to downregulated PKD1-mRNA and increased levels of miRNAs of the miR-200 family. Also, an increase in fibrosis and the recruitment of macrophages due to increased expression of several factors like fibronectin, collagen 1, or cytokines was observed. Upon treatment of cells and mice with an inhibitor of a key enzyme for EV biogenesis, a delay of the disease progression was seen (decreased cyst growth, renal fibrosis, and macrophage populations) [22].

Other studies utilized exo-mRNA as well as exosomal surface proteins to assess the success of kidney transplantations. El Fekhi et al. identified an exo-mRNA signature to discriminate biopsy samples from patients with any-cause rejection of kidney transplants and those with no rejection with an AUC of 0.93 showing a diagnostic improvement compared to the AUC of 0.57 of the current standards of care. They used a urine exosome isolation kit for exosome extraction [24]. Dimuccio et al. analyzed urinary exosomes of healthy volunteers and transplanted patients which they isolated using centrifugation and identified through cytofluorimetric and WB analyses as well as NTA. They observed that levels of the exosomal surface protein CD133, which is decreased in end-stage renal disease, were elevated upon successful kidney transplantation with decreased graft-versus-host-reactions [21].

#### Exosomes as treatment option in nephro- and urological conditions

Exosomal contents that promote kidney injury could be used as targets for delaying the progression of renal diseases or even prevent their formation [22, 44, 53]. Alternatively, protective EVs such as EVs derived from mesenchymal stem cells (MSCs), which are characterized by their regenerative potential could be used for therapy of nephrological diseases. Multiple preclinical studies have shown that EVs (including MSC-derived EVs) display protective effects in kidney injury by promoting regeneration of epithelia, angiogenesis, and reduction of inflammation and apoptosis [8]. Studies on renal fibrosis showed that human umbilical cord MSC-derived exosomes ameliorated the fibrotic phenotype in unilateral ureteral obstruction (UUO) rat models by inhibiting profibrotic pathways and proteins (YAP, p38MAP/ERK) [43, 58]. Even when EVs are derived from other sources, such as endothelial colony forming cells (ECFCs), these have also shown protective effects in ischemic kidney injury (e.g. by exo-miR-486-5p) [98].

The translation of these findings into clinical practice remains a challenge due to a lack of standardized protocols and the difficult distinction of EV-subtypes, their respective functions and characteristics [8].

### Urooncology

Oncosomes, which are present in body fluids at high concentrations [114], have been described extensively as structures of prognostic and diagnostic value [14, 34]. They can be extracted and analyzed regarding their embedded nucleic acids and proteins by performing liquid biopsies which have previously been used as a less invasive tool for the detection of biomarkers like circulating tumor cells (CTCs) [56].

Typically, different diagnostic approaches compete, although the integration of different tests may improve diagnostic performances in cases where combined approaches complement each other. For instance, de la Calle et al. tested a prostate cancer screening algorithm based on liquid biopsies followed by multiparametric MRI (mpMRI) prior to tissue biopsy which aimed at reducing unnecessary biopsies, mpMRI, and over-detection of ISUP (International Society of Urological Pathology) 2014 grade group (GG) 1 prostate cancer (PC) [49].

#### Renal cell carcinoma

Renal cell carcinoma (RCC) makes up about 3% of all cancers worldwide [11]. Late detection in an advanced stage is associated with poor prognosis and stresses the need for identification of biomarkers for early diagnosis [82]. EVs and miRNAs, especially exo-miRs, are the subjects of current research that aims at implementing liquid biopsies as a noninvasive tool for diagnosis, analysis, and monitoring of cancer [74], including clear cell RCC (ccRCC).

Recently, some independent studies demonstrated that specific exo-miR might serve as promising agents for the diagnosis and monitoring of RCC [56, 100, 108] (NCT04053855). Wang et al. and Zhang et al. found that the expression of serum-derived circulating exo-miR-210 in patients with ccRCC was significantly elevated which was associated with metastasis and poor prognosis, and in turn levels significantly decreased within the first week after surgical tumor resection. They isolated exosomes using the Total Exosome Isolation Reagent (Invitrogen) and the Total Exosome Isolation Reagent (Invitrogen) in combination with the EpCAM isolation beads (Invitrogen) to isolate exosomes [100] and EpCAM (epithelial cell adhesion molecule) positive exosomes [108] respectively. Also, Li et al. are currently performing a pilot feasibility study (PEP-C-study, NCT04053855) for molecular detection of RCC via urinary exosomal carbo anhydrase 9 (CA9) [55]. A well-established commercial isolation kit for the isolation and characterization of exosomes is supposed to be used, as well as TEM, flow cytometry and RT-qPCR. If the pilot study is successful, a multicenter study will be conducted.

In addition to the potential significance of exosomes for early diagnosis of RCC, there is evidence that exosomes might help elucidate mechanisms of disease progression and metastasis.

When it comes to systemic therapy, immune checkpoint inhibition (ICI) with tyrosine kinase-inhibitors (TKI) as well as multikinase inhibitors (MKI) [59], such as sunitinib, primary or secondary drug resistance is an issue [31]. A recent study identified lncARSR as an inductor of sunitinib resistance in initially sensitive RCC cells. This was partly due to transport through EVs and could be reversed upon targeting lncARSR [52]. Several substances have already been investigated regarding their inhibiting effects on exosome biogenesis and secretion in drug resistant cancers. These include ketoconazole (KTZ) and tipifarnib (tipi) which have been tested in RCC cell lines or metastatic PC cell lines [18, 34, 35].

Other treatment options targeting EVs or EV-related processes have been suggested upon investigation of RCC progression, especially of metastasis. Zhang et al. found that exo-miR-21-5p derived from M2 macrophages has pro-metastatic effects (in vitro and in vivo) in RCC by activation of the PTEN/Akt pathway. Inhibition of miR-21-5p in M2 exosomes led to a reduction of the metastatic potential of RCC cells. Exosomes were isolated via centrifugation and UC and subsequently identified using TEM and Dynamic Light Scattering (DLS) [111]. In contrast, Li et al. observed that metastatic spread and tumor growth was decreased in an orthotopic mouse model of ccRCC after injection of MSC-derived exosomes which were extracted using UC and identified via NTA, WB analysis and TEM. These effects were attributed to the MSC-derived exo-miR-182 which appeared to promote the T-cell modulated immune response and led to a reduced expression of VEGF-A and overall tumor progression [54].

#### Urothelial carcinoma

The urothelial carcinoma of the bladder (BC) is the 10th most diagnosed cancer among adults [79]. The 5-year progression rate of initially non-muscle-invasive cancer (NMIBC) to muscle-invasive disease (MIBC) with poor prognosis ranges up to 45% [3].

Furthermore, cystoscopy is mandatory for diagnosis of BC. With a sensitivity of 84% for high-grade tumors, urinary cytology serves as an adjunct with insufficient sensitivity for low-grade tumors [105] and negative urinary cytology does not exclude tumor presence. Several potential urinary tests have been investigated [88], but none have been established in the routine clinical workflow which is why the search for molecular biomarkers is ongoing. For instance, Wen et al. performed a study to investigate the potential of exo-CA9 mRNA, which has previously been described as a diagnostic tool for BC [64]. Exosomes were extracted from urine samples of BC patients and controls using a combination of centrifugation, and a urinary exosome isolation solution by Hope Tech Biotechnology and TEM and flow cytometry was performed for the characterization of isolated exosomes. The average level of urinary exosomal CA9 was not only significantly increased in BC patients compared to controls, but Wen et al. also suggest that the detection of exo-CA9 mRNA might have a sufficient sensitivity and specificity to be used as a marker for the diagnosis of BC [102].

Until now, therapy of BC remains challenging due to its heterogeneous tumor biology. Radical cystectomy (RC) is the standard treatment for organ confined MIBC [91], but there is a recurrence rate of up to 40% after RC [87] which is possibly due to early micro-metastatic dissemination or “metabolic rewiring” after proceeding secretion of bladder derived exosomes. Hiltbrunner et al. detected overexpression of urinary exosomal proteins and tumor metabolism-related oncogenes in BC patients’ urinary samples, despite complete histopathological downstaging due to RC and neoadjuvant chemotherapy (NAC). They used UC to isolate exosomes from urine which they subsequently characterized via flow cytometry, NTA, EM, and mass spectrometry. Contrary to ureteral derived exosomes (without prior tumor contact) which did not exhibit an overexpression of these components, urinary exosomes derived from the bladder revealed a potentially malignant “memory” phenotype, despite histopathological downstaging and could promote cancer dissemination [39].

#### Prostate cancer

PC is the 2nd most frequently diagnosed cancer in men worldwide and is the most frequently diagnosed cancer overall in more than 50% of countries [93]. Differentiating between clinical indolent PC and aggressive organ confined PC is crucial. Due to the risk of overdiagnosis and overtreatment on the one hand and lack of clinically significant localized PC eligible for curative treatment on the other hand, the use of prostate specific antigen (PSA) as a marker for early detection must be chosen wisely and recommendations differ between guidelines [2, 40, 51]. Therefore, several new tests have been introduced to extend diagnostic options.

Currently, only one exosome-based diagnostic test for PC detection has been commercialized. The ExoDx Prostate (IntelliScore) (EPI) test discriminates indolent (benign and GG1) from clinically significant PC (≥ GG2) of men with more than 50 years of age with PSA values in a gray-zone of 2–10 ng/ml presenting for biopsy [68]. The test is based on qPCR signals of the three mRNAs of PCA3, ERG, and SPDEF derived from urinary exosomes. Its clinical performance has been validated in three independent, multi-site, prospective clinical validation studies in the US [63, 67, 68, 95]. Furthermore, a European study utilized the EPI-CE test, a CE-marked in vitro diagnostic (IVD) version specifically developed for the use in European laboratories [48]. Two other exosome-based tests are currently under clinical validation (NCT04100811, NCT03957252). The Sentinel test platform consisting of three different tests (Sentinel PCa, Sentinel CS and Sentinel HG) quantifies the expression levels of hundreds of urinary exo-sncRNAs using Affymetrix GeneChips [99]. The ClarityDx test is based on the prostate-specific membrane antigen, polysialic acid, and ghrelin-growth hormone receptor as detected by microflow cytometry data of plasma-derived exosomes [27].

Despite these multi-center validation trials, various smaller studies have been conducted to find potential biomarkers for PC. Recently, miRNAs isolated from urinary EVs by differential centrifugation have been examined in a cohort of 70 patients for their potential to distinguish between ISUP GG1, GG2, and GG3 patients to allow for active surveillance of PC patients as an alternative to radical treatment. While the NGS analysis of miRNA data showed promising classification performances, results could not be reproduced when NGS quantification was replaced by qPCR quantification [76]. A study by Logozzi et al. showed that PSA-expressing exosomes in plasma, that were extracted via centrifugation, were increased in PC patients compared to BPH and control groups by performing NTA, WB, ELISA and flow cytometry [60]. Almeida et al. extracted urinary EVs of PC patients using centrifugation, which they identified with NTA, EM, WB and RNA analyses. The comparison of total RNA-sequencing of FFPE PC tumor tissue and their paired urinary EV measurements highlighted strong differences in circRNA signals between tumor and EVs and suggests an important role of circRNAs in PC detection and treatment [4].

Treatment of advanced PC is another challenging aspect, especially when it comes to castration resistant PC (CRPC), where initially effective androgen deprivation therapy (ADT) becomes insufficient for disease control [13]. Metastatic CRPC (mCRPC) is associated with poor survival and treatment options are limited, although new agents are continuously emerging on the market. Exosomes derived from PC cells and other cells of the TME can promote metastatic spread [1]. Recently, Guo et al. demonstrated that the upregulated lncRNA LINC01213 in exosomes derived from androgen-independent PC (AIPC) cells via UC could induce androgen independency by uptake of former dependent PC cells (ADPC) through activation of the Wnt/β-catenin signaling pathway [36]. These findings introduce exosome contents as well as exosome biogenesis and secretion as new structures of interest for treatment of mCRPC. For instance, Datta et al. have identified Manumycin-A (MA) as a suppressor of exosomal biogenesis in CRPC cells [17]. Del Re et al. demonstrated the detection of androgen receptor splice variant 7 (AR-V7) in plasma-derived exosome RNA as a reliable predictor for hormonal therapy in mCRPC patients. Overall, they view exosome-derived RNA as a more promising diagnostic agent than more invasive and complex procedures such as the extraction of CTCs [19].

## Concluding remarks

Exosome-based research has seen a large increase in interest over the last few years. This is not only due to the previously underestimated importance of exosomes in biological and pathological functions but also attributed to the therapeutic and diagnostic possibilities that arise from the exosomal structure and stability. So far, exosome-based research is still mostly limited to preclinical experiments and early-stage clinical trials, because the translation of experimental findings remains difficult. Even though there are attempts at implementing a standardized protocol for the isolation, purification, and characterization of exosomes by the ISEV, the commonly used methods display disadvantages that make it challenging to utilize them in clinical practice. Although the application of exosomes in diagnostics and clinical therapy is still in its early stages, their importance in physiological and pathophysiological processes appears to be significant and has been observed in a variety of studies.

## Data Availability

Data sharing not applicable to this article as no datasets were generated or analyzed in this review.
